# Risk of Tooth Loss After Cigarette Smoking Cessation

**Published:** 2006-09-15

**Authors:** Elizabeth Krall Kaye, Thomas Dietrich, Martha E Nunn, Raul I Garcia

**Affiliations:** Department of Health Policy and Health Services Research, Boston University Goldman School of Dental Medicine, Veterans Affairs Normative Aging Study and Dental Longitudinal Study, VA Boston Healthcare System; Department of Health Policy and Health Services Research, Boston University Goldman School of Dental Medicine, Veterans Affairs Normative Aging Study and Dental Longitudinal Study, VA Boston Healthcare System, Boston, Mass; Department of Health Policy and Health Services Research, Boston University Goldman School of Dental Medicine, Veterans Affairs Normative Aging Study and Dental Longitudinal Study, VA Boston Healthcare System, Boston, Mass; Department of Health Policy and Health Services Research, Boston University Goldman School of Dental Medicine, Veterans Affairs Normative Aging Study and Dental Longitudinal Study, VA Boston Healthcare System, Boston, Mass

## Abstract

**Introduction:**

Little is known about the effect of cigarette smoking cessation on risk of tooth loss. We examined how risk of tooth loss changed with longer periods of smoking abstinence in a prospective study of oral health in men.

**Methods:**

Research subjects were 789 men who participated in the Veterans Administration Dental Longitudinal Study from 1968 to 2004. Tooth status and smoking status were determined at examinations performed every 3 years, for a maximum follow-up time of 35 years. Risk of tooth loss subsequent to smoking cessation was assessed sequentially at 1-year intervals with multivariate proportional hazards regression models. Men who never smoked cigarettes, cigars, or pipes formed the reference group. Hazard ratios were adjusted for age, education, total pack-years of cigarette exposure, frequency of brushing, and use of floss.

**Results:**

The hazard ratio for tooth loss was 2.1 (95% confidence interval [CI], 1.5–3.1) among men who smoked cigarettes during all or part of follow-up. Risk of tooth loss among men who quit smoking declined as time after smoking cessation increased, from 2.0 (95% CI, 1.4–2.9) after 1 year of abstinence to 1.0 (95% CI, 0.5–2.2) after 15 years of abstinence. The risk remained significantly elevated for the first 9 years of abstinence but eventually dropped to the level of men who never smoked after 13 or more years.

**Conclusion:**

These results indicate that smoking cessation is beneficial for tooth retention, but long-term abstinence is required to reduce the risk to the level of people who have never smoked.

## Introduction

Tooth loss is a persistent health problem among U.S. adults. In the National Health and Nutrition Examination Survey (NHANES) of the U.S. population from 1999 to 2002, individuals in the 45- to 59-year age bracket had an average of 24 teeth remaining (1). As this segment of the population ages, more teeth will be lost because of periodontal disease and caries. Cigarette smoking is a significant risk factor for periodontal disease and accounts for more than half the cases in the population (2). Some studies also suggest that smoking increases the risk of caries (3-5). Previous studies indicate that cigarette smokers are also more likely to have missing teeth (5-8) and experience greater rates of tooth loss (8-15) than nonsmokers. Former smokers retain more teeth than current smokers (7,11,16) but still seem to be at elevated risk of tooth loss relative to people who never smoked (11).

Since the first surgeon general's report on smoking and health detailed the harmful effects of cigarettes on various health conditions (17), many adults have successfully quit smoking. The desire to improve one's health or the health of family members ranks high as a motivation for people to quit smoking (18,19) and to maintain long-term abstinence (20). That motivation may be enhanced if individuals are made aware that their risks of serious chronic diseases can be lowered to the level of someone who never smoked if they remain abstinent long enough. There is variability, however, in the amount of time necessary for risks of different diseases to decline significantly after smoking cessation. The decline in lung function among smokers begins to reverse within a year of cessation (21). However, an increased risk of lung cancer persists for 30 years after quitting among men who were light smokers (<10 cigarettes per day) and for more than 40 years among heavy smokers (22). It is estimated that mortality rates from coronary artery disease and stroke approach those of nonsmokers approximately 15 years after smoking cessation (23), but total mortality and cancer mortality rates among men remain elevated for at least 20 years after quitting (24). It is not known how tooth loss compares with these other chronic diseases. The purpose of this study was to examine whether risk of tooth loss returns to the level of nonsmokers after smoking cessation and, if so, how much time must elapse before this occurs.

## Methods

### Subjects

The Veterans Administration Dental Longitudinal Study (DLS) is a prospective study of oral health and aging in men (25) that has been ongoing since 1968. The DLS initially enrolled 1231 medically healthy men, aged 21 to 84 years, who also were participants in the Normative Aging Study (26). The men were not patients of the U.S. Department of Veterans Affairs (VA) health care system; they received dental and medical care from the private sector. Participants returned to the study site approximately every 3 years for clinical dental examinations and radiographs and to answer questions about dental care and lifestyle. Up to 35 years of follow-up data are included in this analysis. The study was reviewed and approved by the VA Subcommittee on Human Studies and the Boston University Medical Center Institutional Review Board. All participants gave written informed consent.

Of the 1231 men initially enrolled in the DLS, 789 were eligible for this analysis. Exclusion criteria were edentate status at baseline (n = 73), no follow-up examinations after baseline (n = 112), and smoked cigar or pipe at baseline or during follow-up (n = 257).

The 789 eligible participants were grouped into men who had never smoked tobacco (cigarettes, pipes, or cigars) either before baseline or during the study (*never smokers*, n = 264), men who smoked cigarettes before baseline but not during follow-up (*former smokers*, n = 283), or men who smoked cigarettes at the study baseline (*current smokers*, n = 242). The current smokers were further divided into those who subsequently quit smoking and abstained from any type of tobacco product (*quitters*, n = 129) and those who continued to smoke cigarettes at each examination (*continuous smokers*, n = 113). The total length of time smoked was computed from the age participants first smoked to their age at the last DLS examination date at which they reported using cigarettes (quitters) or age at the last DLS examination date they attended (continuous smokers).

### Examinations

At each examination, the number of teeth remaining was counted, and each tooth was evaluated for restorations and caries, probing pocket depth at six sites, and calculus. Probing pocket depth and calculus were recorded as ordinal scores. Pocket depth scores ranged from 0 (≤2 mm) to 3 (≥5 mm), and calculus scores from 0 (none) to 3 (circumferential band around tooth). Alveolar bone loss was measured from periapical radiographs on the distal and mesial sites of each tooth. A modified Schei ruler method (27), which expresses the reduction in alveolar bone height as the percentage of the total distance between the cemento-enamel junction and root apex, was used to score bone loss. The maximum probing pocket depth and bone loss scores per tooth were used in analyses. Because exact dates of tooth loss were unknown, the date of loss was systematically computed as the midpoint between the first DLS examination date at which the tooth was recorded as absent and the examination immediately preceding it.

Educational level and smoking history were obtained by interviewer-administered questionnaires. Information on type of tobacco product used, number of cigarettes smoked per day, and years since last smoked, if applicable, was updated at each examination (28). Participants were first asked about dental insurance coverage in 1987; this information was available for 470 participants, who were categorized as either ever or never having had dental insurance.

### Statistical analysis

Characteristics of the men by smoking status were compared with Kruskal-Wallis one-way analysis of variance (continuous variables) or Χ^2^ statistic (categorical variables). Differences were considered statistically significant at *P* < .05.

Risk of incident tooth loss was estimated in tooth-specific analyses with multivariate proportional hazards regression models using the marginal approach. For never smokers and current smokers, the baseline for follow-up was study enrollment from 1968 to 1973. For the group of quitters, the last examination at which they reported using cigarettes was substituted for their baseline so that only teeth lost after these participants quit smoking were included, and length of abstinence was computed as the amount of time from this new baseline to the date of tooth loss or the last examination date. To describe how risk changed by increasing length of abstinence, we reassessed the proportional hazards in 1-year increments from baseline up to 15 years (the median length of abstinence) in separate models. For example, the model for risk at baseline (0 years of abstinence) included all teeth present at baseline; the model for risk after 1 year of abstinence included only teeth present at 1 year postbaseline; the model for risk after 2 years of abstinence included only teeth present at 2 years postbaseline, and so on. Each regression model contained variables for education (a nine-level variable ranging from grade school to professional degree) and the appropriate age, total pack-years of cigarette exposure (average number of packs smoked per day multiplied by total number of years smoked), frequency of tooth brushing (≤ once per day or > once per day), and use of floss (ever or never) at the particular time frame. Never smokers were the reference group in all models. We determined that the proportional hazards assumption was met by examining log-minus-log plots. The model Χ^2^ statistic in each model was significant at *P* < .001. Hazard ratios (HRs) and 95% confidence intervals (CIs) are presented.

## Results

Characteristics of the men at baseline by smoking status are shown in Table 1. Current cigarette smokers were younger, had the most teeth with greater than 20% alveolar bone loss, and the highest calculus scores. Brushing, flossing and advanced education tended to be underrepresented in current smokers, but these differences were not statistically significant. Among former smokers, the number of teeth remaining and teeth with probing pocket depth greater than 3 mm were similar to current smokers, but number of teeth with alveolar bone loss greater than 20% and calculus score were intermediate to never smokers and current smokers. Among men who were current smokers at baseline, there were no differences in any characteristic between those who went on to quit cigarettes and those who continued to smoke.

Men who continually smoked cigarettes contributed the fewest years of follow-up and had the highest unadjusted rate of tooth loss per 1000 teeth at risk, followed by men who quit cigarettes during the study (Table 2).

**Figure 1:**
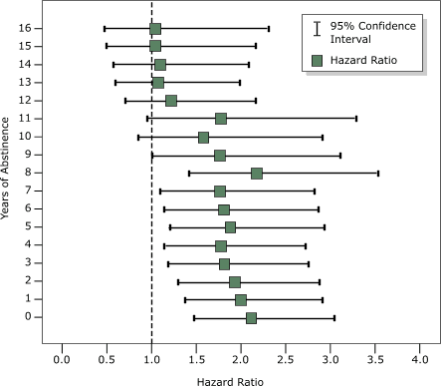
Hazard ratios and 95% confidence intervals for tooth loss among men who quit smoking cigarettes, by years of abstinence, in the Veterans Administration Dental Longitudinal Study, 1968–2004. Each hazard ratio was estimated from separate tooth-specific, multivariate proportional hazards regression models using the marginal approach and was adjusted for education (nine levels ranging from grade school to professional degree), age, total pack-years of cigarette exposure (average number of packs smoked per day multiplied by total number of years smoked), frequency of tooth brushing (≤ once per day or > once per day), and use of floss (ever or never). Never smokers are the reference group; their risk (1.0) is indicated by the dotted line.

**Figure 2:**
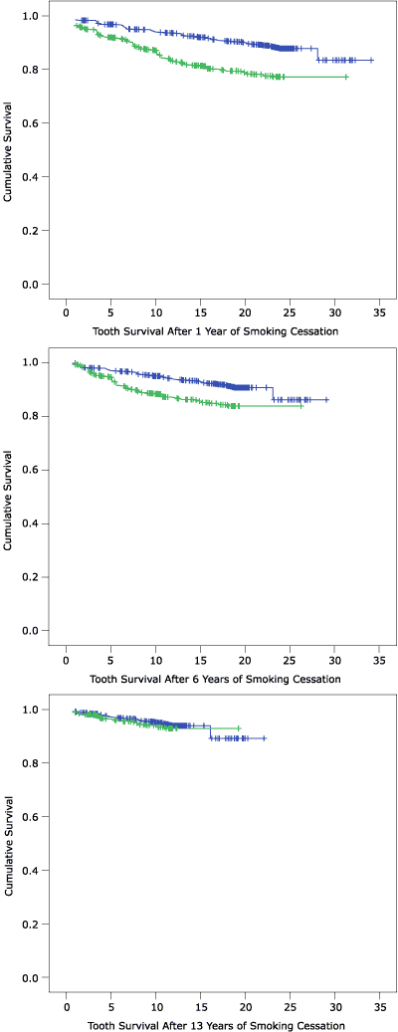
Kaplan-Meier survival plots for teeth in never smokers (blue markers) and quitters (green markers) after 1 year (top), 6 years (middle), and 13 years (bottom) of cigarette abstinence, Veterans Administration Dental Longitudinal Study, 1968–2004.

At baseline, the adjusted HR for tooth loss was 2.1 (95% CI, 1.5–3.1) among all men who smoked cigarettes, and 1.3 (95% CI, 0.9–1.7) among former smokers. HRs among men who quit smoking declined after they became abstinent but remained significantly elevated above the level of never-smokers until 9 years of abstinence had passed (Figure 1). At 13 years of abstinence and beyond, the HRs approached and stayed very close to 1.0. Tooth survival plots comparing quitters and never-smokers after 1, 6, and 13 years of abstinence are shown in Figure 2.

**Table:** 

**Years of Abstinence**	**Hazard Ratio (95% Confidence Interval)**
0	2.1 (1.5 – 3.1)
1	2.0 (1.4 – 2.9)
2	1.9 (1.3 – 2.9)
3	1.8 (1.2 – 2.8)
4	1.8 (1.2 – 2.7)
5	1.9 (1.2 – 2.9)
6	1.8 ( 1.1 – 2.9)
7	1.8 (1.1 – 2.8)
8	2.2 (1.3 – 3.6)
9	1.8 (1.0 – 3.1)
10	1.6 (0.9 – 2.9)
11	1.8 (1.0 – 3.3)
12	1.2 (0.7 – 2.2)
13	1.1 (0.6 – 2.0)
14	1.1 (0.6 – 2.0)
15	1.0 (0.5 – 2.2)
16	1.0 (0.5 – 2.3)

Because dental insurance information was available for only 60% of participants, two models for risk at 0 years of abstinence were constructed in the subset of men with valid insurance data, one which included only the independent variables listed above and one which also included insurance (ever or never). The HRs and CIs were identical for current smokers (HR, 2.1; 95% CI, 1.3–3.3) and former smokers (HR, 1.3; 95% CI, 0.9–1.8) whether insurance was included or excluded.

## Discussion

Sufficient evidence has accumulated to conclude that smoking is a causal factor in cardiovascular diseases, certain types of cancer, chronic obstructive lung diseases, infertility, cataracts, hip fractures, and periodontal diseases (29). Risks of some of these diseases decline when the causal factor is removed through smoking cessation (30). Research suggests smoking also may be causally linked to tooth loss (8-15), but there is little information on the effect of smoking cessation on tooth loss risk.

A previous analysis of DLS participants found that the rate of tooth loss among men who quit smoking was about 50% lower than the rate among current smokers but still significantly higher than the rate among nonsmokers (8). However, that analysis did not address how risk might change with increasing length of abstinence. In a 12-year follow-up study of 1031 Swedish women, prospective rates of tooth loss were similar in never smokers and former smokers who had abstained from smoking an average of 10 years before entering the study (13). These findings are consistent with the arrested progression of periodontal bone loss and attachment loss observed when individuals quit smoking (31-33). 

The results of this study suggest that tooth loss risk does decline after smoking cessation but that the risk remains elevated in relation to nonsmokers for at least 9 years. Why should the risk of tooth loss decline as men remain abstinent from cigarettes, and why does it seem to take about a decade or more to return to the level of never-smokers? The loss of alveolar bone is not reversible, so one might expect the cumulative damage to the bone tissue by cigarettes to keep the risk of tooth loss permanently elevated. But periodontal disease is often localized around a few teeth, as demonstrated by the small number of teeth with moderate alveolar bone loss and probing pocket depths in this cohort, and progresses intermittently. Removing exposure to smoke reduces the likelihood that disease will become widespread and affect many teeth. In addition, smoking is one of several risk factors for periodontal disease. Age, genetic susceptibility, and systemic diseases such as diabetes all influence the disease risk. It may be that as time elapses, these other risk factors become more important and begin to obscure the differences due to past smoking. Finally, there are other lifestyle changes that may occur when an individual decides to quit smoking and may become more established as the duration of abstinence increases. Smokers who quit appear to be more health conscious than those who continue to smoke, and they make physician visits and use health screening programs at rates comparable to those of nonsmokers (34). Former smokers in the DLS were more likely than current smokers to have had a dental prophylaxis in the past year (8), a practice that should promote tooth retention rather than tooth loss.

The risk of tooth loss in quitters was not significantly different from that in never smokers more than 9 years after cessation and remained consistently near 1.0 after 13 years. The length of time needed to lower the risk to the level of never smokers could not be determined more accurately in our subject population. The number of subjects decreased, and the confidence intervals widened, as the baseline used to compute tooth survival was moved forward to account for increasing periods of abstinence. Nevertheless, the data suggest that the length of time after smoking cessation needed to significantly lower risk is not so long as to be unattainable yet requires long-term commitment to avoid smoking relapse.

This study has several limitations that could affect our estimates of tooth loss risks and of when the risk for quitters reaches the level of never smokers. Information on the causes of tooth loss was not obtained. We assume that teeth were lost primarily because of periodontal disease or caries, but it is possible that some teeth were extracted for other reasons unrelated to these diseases. There may have been confounding by education, socioeconomic status, and dental insurance coverage that we could not control for adequately. Although we had some information on these measures, it was not necessarily complete. Education was recorded as a nine-level categorical variable rather than years completed. Socioeconomic status (income) was assessed only at the study baseline and was not updated during follow-up, even though the employment status of the men changed. Dental insurance information was not obtained until almost 20 years had elapsed since baseline and therefore was missing for the 40% of the cohort that had dropped out by this time. It is possible that insurance coverage of smokers who dropped out early was different from that of smokers who remained in the study. In addition, the study included only men and few individuals from minority populations. Therefore, the ability to generalize these results to different populations is limited.

The results of this study suggest that the risk of tooth loss decreases upon smoking cessation, but it may take at least 9 to 12 years of abstinence for the risk to return to the level of never smokers. This information can be used to encourage current smokers to quit and to remain abstinent.

## Figures and Tables

**Table 1:** Characteristics of Men by Cigarette Smoking Status at Baseline, Veterans Administration Dental Longitudinal Study

**Characteristic**	**Never Smokers (n = 264)**	**Former Smokers (n = 283)**	**Current Smokers (n = 242)**	***P *Value^a^**
Age in years, mean (SD)	50 (10)	49 (9)	45 (8)	<.001
No. of teeth, mean (SD)	24 (6)	23 (6)	23 (6)	.008
No. of decayed or filled surfaces per tooth, mean (SD)	1.6 (0.8)	1.7 (0.7)	1.6 (0.7)	.18
No. of teeth with >20% alveolar bone loss^b^	0 (0,3)	1 (0,4)	2 (0,7)	<.001
No. of teeth with probing pocket depth >3 mm^b^	2 (0,5)	3 (0,6)	3 (1,8)	<.001
Average calculus score, mean (SD)	1.2 (0.7)	1.3 (0.7)	1.7 (0.8)	<.001
Subjects brushing >1 time per day, %	44	45	41	.69
Subjects ever floss, %	35	30	31	.19
Subjects ever had dental insurance, % (no. with valid data)^c^	53 (183)	48 (187)	56 (127)	.14
Educational level, %				
College graduate	35	29	23	.08
Technical degree or some college	27	30	28	
High school only	29	31	39	
Did not finish high school	9	10	10	

a *P* value for differences between groups determined from Kruskal-Wallis test (age, number of teeth present, decayed or filled surfaces, number of teeth with alveolar bone loss >20%, number of teeth with probing depth >3mm, and average calculus score) or Χ^2^ test (brush, floss, insurance, and education).

b Median, with 25th and 75th percentiles shown in parentheses.

c Insurance coverage was first assessed in 1987.

**Table 2:** Cigarette Smoking and Tooth Loss by Smoking Status During Follow-up, Veterans Administration Dental Longitudinal Study, 1968–2004

**Characteristic**	**Never Smokers (n = 264)**	**Former Smokers (n = 283)**	**Quitters^a^ (n = 129)**	**Continuous Smokers^b^ (n = 113)**
Total pack-years of exposure^c^	NA	17 (7,30)	26 (19,19, 31)	39 (21,57)
Years of follow-up, mean (SD)	23 (9)	22 (10)	23 (9)	13 (9)
No. of teeth lost per person^c^	1 (0,3)	1 (0,4)	3 (1,8)	2 (0,4)
No. of teeth lost per year per 1000 teeth at risk^c^	2 (0,7)	3 (0,11)	7 (2,20)	8 (0,17)

NA indicates not applicable.

a Participants who were current smokers at baseline and who subsequently quit smoking and abstained from any type of tobacco product.

b Participants who were current smokers at baseline and who continued to report being current smokers at each examination.

c Median, with 25th and 75th percentiles shown in parentheses.
